# METTL16 promotes hepatocellular carcinoma progression through downregulating RAB11B-AS1 in an m^6^A-dependent manner

**DOI:** 10.1186/s11658-022-00342-8

**Published:** 2022-05-20

**Authors:** Yun-zhang Dai, Yong-da Liu, Jie Li, Mei-ting Chen, Mei Huang, Fang Wang, Qing-song Yang, Ji-hang Yuan, Shu-han Sun

**Affiliations:** 1 Department of Medical Genetics, Naval Medical University, Shanghai, 200433 China; 2 Department of Interventional Radiology, Changhai Hospital, Naval Medical University, Shanghai, 20043 China

**Keywords:** Hepatocellular carcinoma, *N*^6^-methyladenosine, RNA methyltransferase, Long noncoding RNA, Tumor progression

## Abstract

**Background:**

The molecular mechanisms driving hepatocellular carcinoma (HCC) remain largely unclear. As one of the major epitranscriptomic modifications, *N*^6^-methyladenosine (m^6^A) plays key roles in HCC. The aim of this study was to investigate the expression, roles, and mechanisms of action of the RNA methyltransferase methyltransferase-like protein 16 (METTL16) in HCC.

**Methods:**

The expression of METTL16 and RAB11B-AS1 was determined by RT-qPCR. The regulation of RAB11B-AS1 by METTL16 was investigated by RNA immunoprecipitation (RIP), methylated RIP (MeRIP), and RNA stability assays. In vitro and in vivo gain- and loss-of-function assays were performed to investigate the roles of METTL16 and RAB11B-AS1.

**Results:**

METTL16 was upregulated in HCC, and its increased expression was correlated with poor prognosis of HCC patients. METTL16 promoted HCC cellular proliferation, migration, and invasion, repressed HCC cellular apoptosis, and promoted HCC tumoral growth in vivo. METTL16 directly bound long noncoding RNA (lncRNA) RAB11B-AS1, induced m^6^A modification of RAB11B-AS1, and decreased the stability of RAB11B-AS1 transcript, leading to the downregulation of RAB11B-AS1. Conversely to METTL16, RAB11B-AS1 is downregulated in HCC, and its decreased expression was correlated with poor prognosis of patients with HCC. Furthermore, the expression of RAB11B-AS1 was negatively correlated with METTL16 in HCC tissues. RAB11B-AS1 repressed HCC cellular proliferation, migration, and invasion, promoted HCC cellular apoptosis, and inhibited HCC tumoral growth in vivo. Functional rescue assays revealed that overexpression of RAB11B-AS1 reversed the oncogenic roles of METTL16 in HCC.

**Conclusions:**

This study identified the METTL16/RAB11B-AS1 regulatory axis in HCC, which represented novel targets for HCC prognosis and treatment.

**Supplementary Information:**

The online version contains supplementary material available at 10.1186/s11658-022-00342-8.

## Background

Liver cancer is the second leading cause of cancer-related death worldwide [1]. Hepatocellular carcinoma (HCC) is the major histological type of liver cancer [2]. The initiation and progression of HCC involve many genetic and epigenetic alterations [3–6]. Recently, aberrantly epitranscriptomic modifications (posttranscriptional chemical modifications) of RNAs have been identified in human diseases [7, 8]. As one of the most prevalent chemical modifications of RNA, *N*^6^-methyladenosine (m^6^A) shows vitally important roles in various pathophysiological processes through modulating the stability, translation, splicing, or functions of target RNAs [9–11]. Aberrant m^6^A modifications are found in a variety of diseases, including HCC [12, 13].

m^6^A modification is reversibly installed by RNA methyltransferases and demethylases [14]. The critical mRNA methyltransferases include METTL3–METTL14 complex, which are also known as m^6^A writers [15]. The critical RNA demethylases include FTO and ALKBH5, which are known as m^6^A erasers [16, 17]. The involvements of METTL3, METTL14, FTO, and ALKBH5 in many cancers have been investigated, and they demonstrate various roles in different cancers [18–20].

METTL16 is a recently identified m^6^A methyltransferase, which has important roles in embryonic development [21]. Although the oncogenic roles of METTL16 in gastric cancer has been reported [22], the role, expression, and clinical relevance of METTL16 in other cancers, including HCC, are still unclear. As an m^6^A writer, METTL16 was reported to directly deposit m^6^A in only MAT2A mRNA and U6 small nuclear RNA (snRNA) [23]. Under low *S*-adenosylmethionine condition, METTL16 was localized at MAT2A mRNA 3′ UTR and induced splicing of the MAT2A retained intron, leading to decreased degradation of MAT2A mRNA [23]. Furthermore, METTL16 was identified to bind several noncoding RNAs and pre-mRNAs, including MAT2A, U6 snRNA, MALAT1, and XIST [24–26]. MALAT1 and XIST are long noncoding RNAs (lncRNAs).

lncRNAs are a class of transcripts that have no protein-encoding potential and are more than 200 nucleotides in length [27, 28]. Transcriptomic sequencing has found more than 58,000 lncRNAs in human cells [29]. However, the number of human mRNAs is only about 21,000. Many lncRNAs were revealed to be dysregulated in various diseases, including HCC [30–34]. Moreover, many lncRNAs show important roles in various pathophysiological processes [35–42]. In cancers, several lncRNAs were revealed to exert oncogenic or tumor-suppressive roles [43–45]. RAB11B-AS1 is a recently reported cancer-related lncRNA, which has various roles in different cancers [46, 47]. RAB11B-AS1 has 1034 nucleotides and three exons. *RAB11B-AS1* is located at chromosome 19p13.2. Whether and which lncRNAs mediate the roles of METTL16 in HCC is still unknown.

In this study, we investigated the expression, role, and mechanism of action of METTL16 in HCC. Using publicly available datasets and our own cohort, we found that METTL16 was upregulated in HCC and associated with poor prognosis of HCC patients. Furthermore, we investigated the biological roles of METTL16 in HCC and identified lncRNA RAB11B-AS1 as the downstream target of METTL16, which mediates the oncogenic roles of METTL16 in HCC.

## Materials and methods

### Cell culture

Human HCC cells HepG2 (cat. no. SCSP-510) and Huh7 (cat. no. SCSP-526) were acquired from the Chinese Academy of Sciences Cell Bank (Shanghai, China) and cultured in Eagle’s Minimum Essential Medium and Dulbecco’s Modified Eagle’s Medium, respectively, with 10% fetal bovine serum (FBS) added. Human HCC cells SNU-398 (cat. no. CRL­2233) were acquired from American Type Culture Collection (ATCC, Manassas, VA, USA) and cultured in RPMI 1640 medium with 10% FBS added. All cells were authenticated using STR profiling and confirmed to be mycoplasma free.

### Tissue samples

A total of 63 pairs of human HCC tissues and matched adjacent noncancerous liver tissues were acquired at the Eastern Hepatobiliary Surgery Hospital affiliated to our university (Shanghai, China). All tissues were confirmed by pathological examination and stored at −80 °C until use. Written informed consent was obtained from all patients. This study was approved by the Committee on Ethics of Biomedicine, Second Military Medical University.

### RNA extraction and quantitative polymerase chain reaction (qPCR)

Total RNA was isolated using the RNA isolater Total RNA Extraction Reagent (cat. no. R401-01, Vazyme, Nanjing, China). The RNA was used to perform reverse transcription (RT) using the HiScript III RT SuperMix for qPCR kit (Cat. R323, Vazyme). Real-time qPCR was undertaken in a StepOnePlus Real-Time PCR System (Applied Biosystems) using the ChamQ Universal SYBR qPCR Master Mix (Vazyme). The sequences of primers were as follows: for METTL16, 5′-AGGGAGTAAACTCACGAAATCCT-3′ (forward), 5′-AACCCCTTGTATGCGAAGCTC-3′ (reverse); for RAB11B-AS1, 5′-GCGAAGCCAATCAGAGATGG-3′ (forward), 5′-CTTGAGCTCGCCCCTGATAG-3′ (reverse); for MAT2A, 5′-AATATTGAAAGTGTTAGCCT-3′ (forward), 5′-AGGAAAATTTAGGAAGGAG-3′ (reverse); for EEF1A1, 5′-CGGTCTCAGAACTGTTTGTTTC-3′ (forward), 5′-AAACCAAAGTGGTCCACAAA-3′ (reverse); for GAPDH, 5′-GGTCTCCTCTGACTTCAACA-3′ (forward), 5′-GTGAGGGTCTCTCTCTTCCT-3′ (reverse). GAPDH was used as an endogenous control for the quantification of mRNAs and lncRNAs. Relative expression was analyzed using the 2^−ΔΔCt^ method.

### Vector construction, small interfering RNA (siRNA) synthesis, and transfection

METTL16-expressing lentivirus (LV17 vector) was purchased from GenePharma (Shanghai, China). The siRNAs specifically targeting METTL16 were purchased from GenePharma. The sequences of siRNAs were 5′-CUUGAGACUCAACUAUAUUTT-3′ (siMETTL16-1) and 5′-GGCUGGUAUUUCCUCGCAATT-3′ (siMETTL16-2). Scrambled nontargeting siRNA was used as negative control (NC). The cDNA encoding RAB11B-AS1 was PCR-amplified using the PrimeSTAR Max DNA Polymerase (cat. no. R045Q, Takara, Dalian, China) and the primers 5′-AGTGTGGTGGAATTCTGCAGATATCGCCCCGGCGCGTCCTAGGTCC-3′ (forward), 5′-TTCGAAGGGCCCTCTAGACTCGAGTTCTTTGTTCTTGTTTGTTTTCTTT-3′ (reverse). The PCR products were cloned into the EcoR V and Xho I sites of the pcDNA3.1 vector (Invitrogen, Carlsbad, CA, USA) using the NovoRec plus One step PCR Cloning Kit (Novoprotein, Suzhou, China) to construct RAB11B-AS1 overexpression vector. The siRNAs specifically targeting RAB11B-AS1 were purchased from GenePharma. The sequences of siRNAs were 5′-GACAGACCAAAUAACUAAUTT-3′ (siRAB11B-AS1-1) and 5′-CCUGGGAACAUGUUUACAUTT-3′ (siRAB11B-AS1-2). The transfection of vectors and siRNAs was undertaken using the GP-transfect-Mate (GenePharma) following the manufacturer’s manual.

### Stable cell line construction

To obtain cell lines stably overexpressing METTL16, SNU-398 and HepG2 cells were infected with METTL16-expressing lentivirus. The infected cells were plated at very low density in a new six-well plate and treated with 5 µg/ml puromycin to pick METTL16-overexpressed single clone cells. The efficiencies of overexpression were detected by western blot. To obtain cell lines stably overexpressing RAB11B-AS1, SNU-398, and HepG2 cells with transfected with RAB11B-AS1 overexpression vector. The transfected cells were treated with 800 µg/ml neomycin to select RAB11B-AS1-overexpressed single clone cells. To obtain cell lines stably overexpressing METTL16 and RAB11B-AS1, METTL16-overexpressed SNU-398 and HepG2 cells were transfected with RAB11B-AS1 overexpression vector. After transfection, the cells were treated with 5 µg/ml puromycin and 800 µg/ml neomycin to select METTL16- and RAB11B-AS1-overexpressed single clone cells.

### Western blot analysis

Total proteins were harvested from indicated cells using RIPA buffer (Beyotime, Shanghai, China). After quantification using the Enhanced BCA Protein Assay Kit (Beyotime), identical quantities of proteins were separated by SurePAGE prefabricated gels (Bis–Tris, 4–20%, cat. no. M00657, GenScript, Nanjing, China), and transferred onto PVDF membranes (Millipore, Billerica, MA, USA). After incubation with primary antibodies against METTL16 (78 kDa, #17676, 1:1000, Cell Signaling Technology, Danvers, MA, USA) or GAPDH (36 kDa, 60004-1-Ig, 1:5000, Proteintech, Chicago, IL, USA) at 4 °C overnight, the membranes were further incubated with IRDye 680RD Goat anti-Mouse IgG Secondary Antibody (926-68070, 1:10000, Li-Cor Biosciences, Lincoln, NE, USA) or IRDye 800CW Goat anti-Rabbit IgG Secondary Antibody (926-32211, 1:10000, Li-Cor). Lastly, the membranes were scanned on an Odyssey infrared scanner (Li-Cor). GAPDH was employed as endogenous control.

### Cell proliferation assay

Cell Counting Kit-8 (CCK-8) and 5-ethynyl-2′-deoxyuridine (EdU) incorporation assays were undertaken to evaluate cell proliferation as we previously described [4]. Briefly, for CCK-8 assay, 1.5 × 10^3^ cells per well were plated into 96-well plate. At the indicated time, 10 µl CCK-8 reagent (Dojindo Laboratories, Kumamoto, Japan) was added to each well. After incubation for another 2 h, absorbance was measured at 450 nm to indicate cell proliferation ability. For EdU incorporation assay, 2 × 10^5^ cells per well were plated into cover slips in 24-well plate and incubated for overnight. EdU incorporation assays were performed using the Cell-Light EdU Apollo567 In Vitro Kit (RiboBio, Guangzhou, China).

### Cell apoptosis assay

Terminal deoxynucleotidyl transferase (TdT)-mediated dUTP nick end labeling (TUNEL) and caspase-3 activity assays were undertaken to evaluate cell apoptosis. TUNEL assay was performed using the TUNEL BrightRed Apoptosis Detection Kit (Cat. A113, Vazyme) strictly following the provided manual. Caspase-3 activity assay was performed using the Caspase 3 Activity Assay Kit (cat. no. C1116, Beyotime) according to the provided protocol.

### Transwell migration and invasion assays

Transwell migration and invasion assays were undertaken to evaluate cell migration and invasion, respectively. Briefly, 3 × 10^4^ cells resuspended in serum-free medium added with 5 µg/ml mitomycin C (Selleck, Houston, TX, USA) to block cell proliferation were placed into the upper chamber of transwell filter inserts (8-μm pore size, Corning, NY, USA) with or without precoated Matrigel (Corning). Complete medium added with 10% FBS was loaded into the lower chambers. After culture for another 36 h, the cells remaining on the upper chamber were removed. The cells migrating or invading into the bottom side of the inserts were fixed, stained, and quantified under a microscope by counting at least five random high-power fields.

### RNA stability assay

To evaluate the stability of RAB11B-AS1 transcript, indicated cells were cultured in six-well plates and treated with 50 µM α-amanitin (Sigma-Aldrich, Saint Louis, MO, USA) for 0, 2, 4, 6, and 8 h. Total RNA was extracted and reversely transcribed. The relative abundance of RAB11B-AS1 was quantified using RT-qPCR (relative to 0 h).

### RNA immunoprecipitation (RIP) and ultraviolet cross-linking immunoprecipitation (CLIP) assays

RIP assay was performed using the EZ-Magna RIP Kit (cat. no. 17-701, Millipore) and primary antibody against METTL16 (5 µg, #17676, Cell Signaling Technology) strictly following the provided protocol. Methylated RNA immunoprecipitation (MeRIP) assay was performed using the Magna MeRIP m^6^A Kit (cat. no. 17-10499, Millipore). CLIP assay was performed following the reported protocol [48]. The enrichment of RNA was measured by RT-qPCR.

### Animal experiments

Five-week-old male athymic BALB/c nude mice were used for animal experiments. SNU-398 cells (5 × 10^6^) were injected subcutaneously into the flanks of mice. After feeding for another 21 days, the subcutaneous tumors were resected and weighed. The investigators recording tumor growth were blinded to mice allocation. The Committee on Ethics of Biomedicine, Second Military Medical University, approved the animal experiments.

### Bioinformatic analysis

METTL16 and RAB11B-AS1 expression levels in Gene Expression Omnibus (GEO) GSE45436 dataset were analyzed using the probe 226744_at for METTL16 and the probe 1556123_a_at for RAB11B-AS1. The correlations between METTL16, RAB11B-AS1 expression, and overall survival in The Cancer Genome Atlas (TCGA) liver hepatocellular carcinoma (LIHC) dataset was analyzed using the online in silico tool Kaplan–Meier Plotter (https://kmplot.com/analysis/index.php?p=service&cancer=pancancer_rnaseq) with the setting of auto-select best cutoff [49]. The correlation between METTL16 and RAB11B-AS1 expression in TCGA LIHC dataset was analyzed using the online in silico tool ENCORI (https://starbase.sysu.edu.cn/panGeneCoExp.php) [50].

### Statistical analysis

All statistical analyses were conducted with the GraphPad Prism 6.0 Software. For comparison, Mann–Whitney test, Wilcoxon signed-rank test, log-rank test (Kaplan–Meier survival analysis), Student’s *t*-test (two-sided), one-way ANOVA followed by Dunnett’s multiple comparisons test, and Spearman correlation analysis were performed as indicated in figure legends. *P* < 0.05 was considered significant.

## Results

### METTL16 was upregulated in HCC and associated with poor outcomes in patients with HCC

**Fig. 1:**
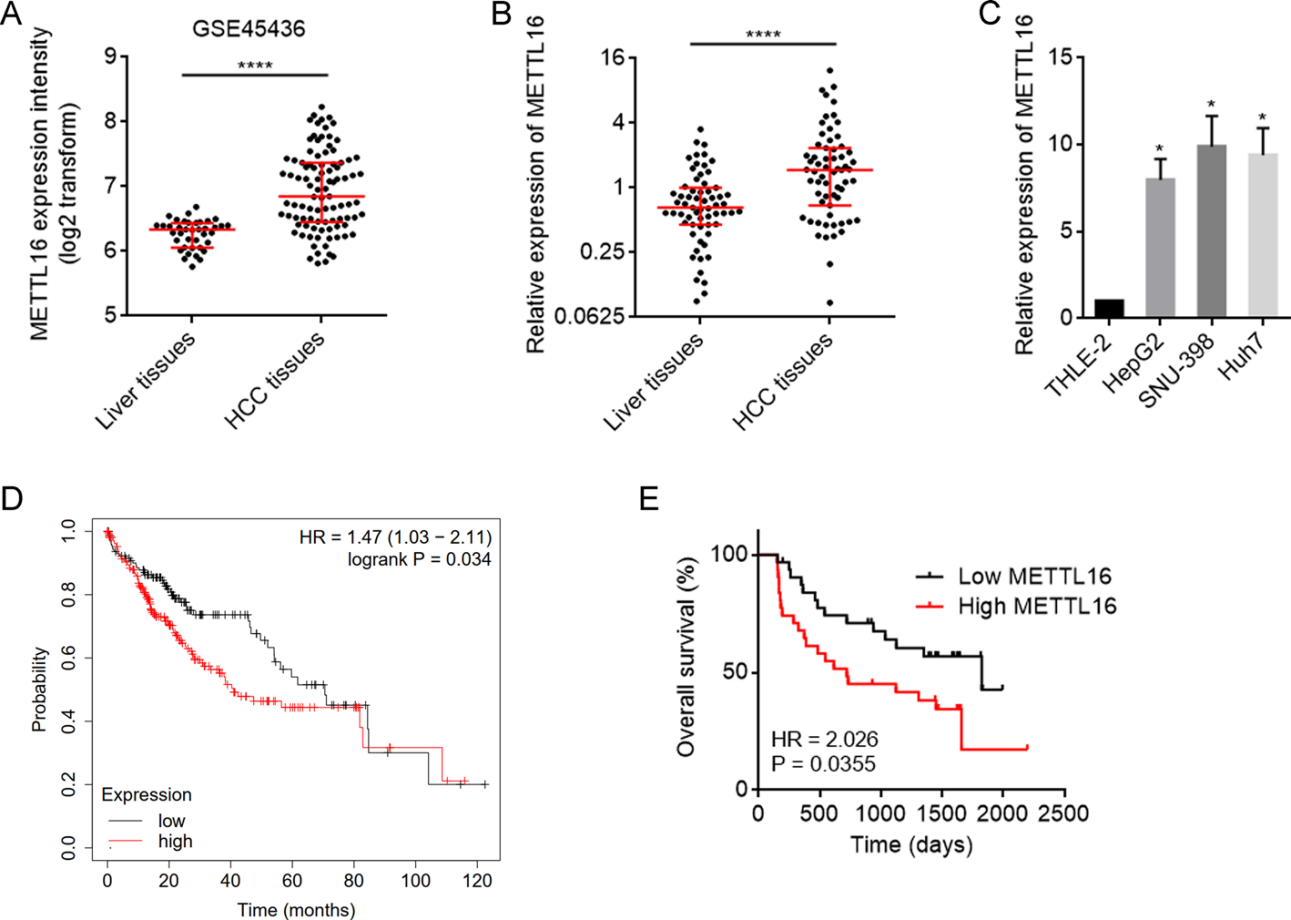
The expression pattern and clinical association of METTL16 in HCC. **A** METTL16 mRNA expression in human HCC tissues (*n* = 95) and normal liver tissues (*n* = 39) from GSE45436 dataset. ****P < 0.0001 by Mann–Whitney test. **B** METTL16 mRNA expression level in 63 pairs of HCC tissues and matched adjacent noncancerous liver tissues was measured by RT-qPCR. ****P < 0.0001 by Wilcoxon signed-rank test. **C** METTL16 mRNA expression level in HCC cell lines HepG2, SNU-398, Huh7, and normal hepatic cell line TLHE-2 was measured by RT-qPCR. Results are shown as mean ± standard deviation (SD) of *n* = 3 independent experiments. *P < 0.05 by one-way ANOVA followed by Dunnett’s multiple comparisons test. **D** Kaplan–Meier survival analysis of the correlation between METTL16 mRNA expression and overall survival based on TCGA liver cancer data, analyzed by the online in silico tool Kaplan–Meier Plotter (https://kmplot.com/analysis/). **E** Kaplan–Meier survival analysis of the correlation between METTL16 mRNA expression and overall survival in our HCC cohort. *n* = 63 patients with HCC. *P* = 0.0355 by log-rank test. Median METTL16 level was used as cutoff

To clarify the expression of METTL16 in HCC, we first analyzed GSE45436 dataset, which contains 39 normal liver tissues and 95 HCC tissues. The results showed that the mRNA level of METTL16 was significantly higher in HCC tissues than in normal liver tissues (Fig. 1A). Furthermore, we collected 63 pairs of HCC tissues and matched adjacent noncancerous liver tissues and measured METTL16 mRNA expression, which also revealed the higher mRNA expression of METTL16 in HCC tissues (Fig. 1B). METTL16 mRNA level was also found to be increased in HCC cell lines HepG2, SNU-398, and Huh7 compared with normal hepatic cell line TLHE-2 (Fig. 1C). To analyze the correlation between METTL16 mRNA expression and prognosis, TCGA dataset was analyzed using the online in silico tool Kaplan–Meier Plotter, which showed that high METTL16 mRNA expression level was significantly correlated with poor overall survival in HCC (Fig. 1D; Additional file 1: Fig. S1). In our cohort, Kaplan–Meier survival analysis also showed that patients with higher METTL16 mRNA level had worse overall survival (Fig. 1E).

### Overexpression of METTL16 exerted oncogenic roles in HCC

**Fig. 2:**
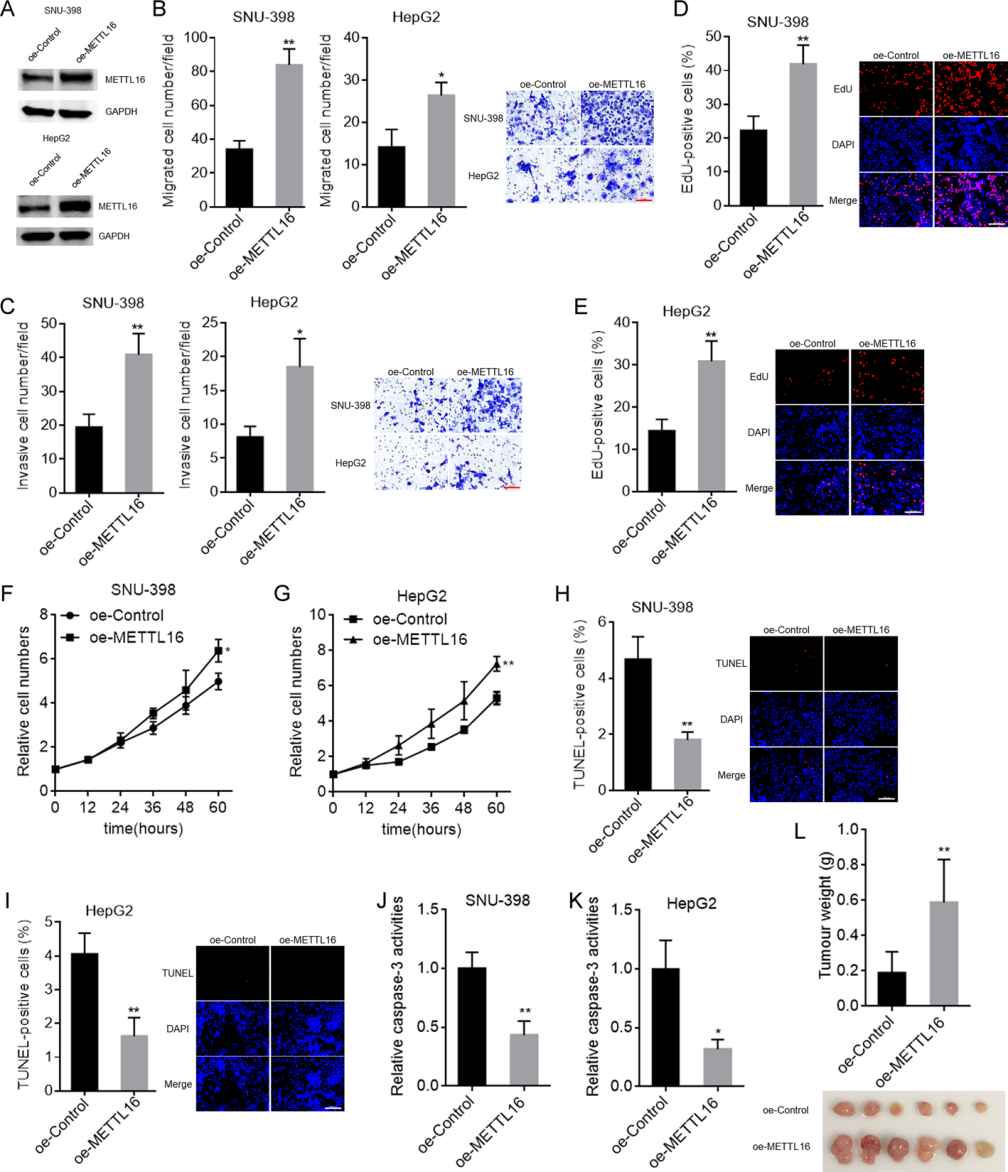
The roles of METTL16 overexpression in HCC. **A** METTL16 protein expression in SNU-398 and HepG2 cells with METTL16 overexpression or control was detected by western blot. **B** Migration ability of SNU-398 and HepG2 cells with METTL16 overexpression or control was detected by transwell migration assay. Scale bars, 100 µm. **C** Invasion ability of SNU-398 and HepG2 cells with METTL16 overexpression or control was detected by transwell invasion assay. Scale bars, 100 µm. **D** Cellular proliferation of SNU-398 cells with METTL16 overexpression or control was detected by EdU assay. Scale bars, 100 µm. **E** Cellular proliferation of HepG2 cells with METTL16 overexpression or control was detected by EdU assay. Scale bars, 100 µm. **F** Cellular proliferation of SNU-398 cells with METTL16 overexpression or control was detected by CCK-8 assay. **G** Cellular proliferation of HepG2 cells with METTL16 overexpression or control was detected by CCK-8 assay. **H** Cellular apoptosis of SNU-398 cells with METTL16 overexpression or control was detected by TUNEL assay. Scale bars, 100 µm. **I** Cellular apoptosis of HepG2 cells with METTL16 overexpression or control was detected by TUNEL assay. Scale bars = 100 µm. **J** Cellular apoptosis of SNU-398 cells with METTL16 overexpression or control was detected by caspase-3 activity assay. **K** Cellular apoptosis of HepG2 cells with METTL16 overexpression or control was detected by caspase-3 activity assay. **L** Weight and photograph of subcutaneous tumors formed by SNU-398 cell with METTL16 overexpression or control. Results are shown as mean ± SD of *n* = 3 independent experiments (**B**–**K**) or *n* = 6 mice in each group (**L**). *P < 0.05, **P < 0.01 by Student’s *t*-test (**B**–**K**) or Mann–Whitney test (**L**)

To further explore the biological roles of METTL16 in HCC, SNU-398 and HepG2 cells with METTL16 stable overexpression were constructed using METTL16 overexpression lentivirus (Fig. 2A). To evaluate the roles of METTL16 overexpression in cellular migration and invasion, transwell migration and invasion assays were carried out. The results showed that METTL16 overexpression increased SNU-398 and HepG2 cellular migration and invasion abilities (Fig. 2B, C). To evaluate the roles of METTL16 overexpression in cellular proliferation, EdU and CCK-8 assays were carried out. EdU assays showed that SNU-398 and HepG2 cells with METTL16 overexpression had increased percentage of EdU-positive cells (Fig. 2D, E), indicating that METTL16 overexpression promoted cellular proliferation. CCK-8 assays also showed that METTL16 overexpression remarkably promoted SNU-398 and HepG2 cellular proliferation (Fig. 2F, G). To evaluate the roles of METTL16 overexpression in cellular apoptosis, TUNEL and caspase-3 activity assays were carried out. TUNEL assays showed that SNU-398 and HepG2 cells with METTL16 overexpression had decreased percentage of TUNEL-positive cells (Fig. 2H, I). Caspase-3 activity assays showed that METTL16 overexpression significantly decreased caspase-3 activity in SNU-398 and HepG2 cells (Fig. 2J, K). Thus, both TUNEL and caspase-3 activity assays suggested that METTL16 overexpression repressed SNU-398 and HepG2 cellular apoptosis. To evaluate the biological roles of METTL16 overexpression in vivo, SNU-398 cells with METTL16 stable overexpression or control were subcutaneously injected into BALB/c nude mice. SNU-398 cells with METTL16 overexpression formed much larger tumors than control SNU-398 cells (Fig. 2L). Collectively, these data demonstrate that METTL16 overexpression promoted HCC cellular migration, invasion, and proliferation, inhibited HCC cellular apoptosis, and promoted HCC tumor growth in vivo, indicating that METTL16 overexpression has oncogenic roles in HCC.

### METTL16 silencing exerted tumor-suppressive roles in HCC

**Fig. 3:**
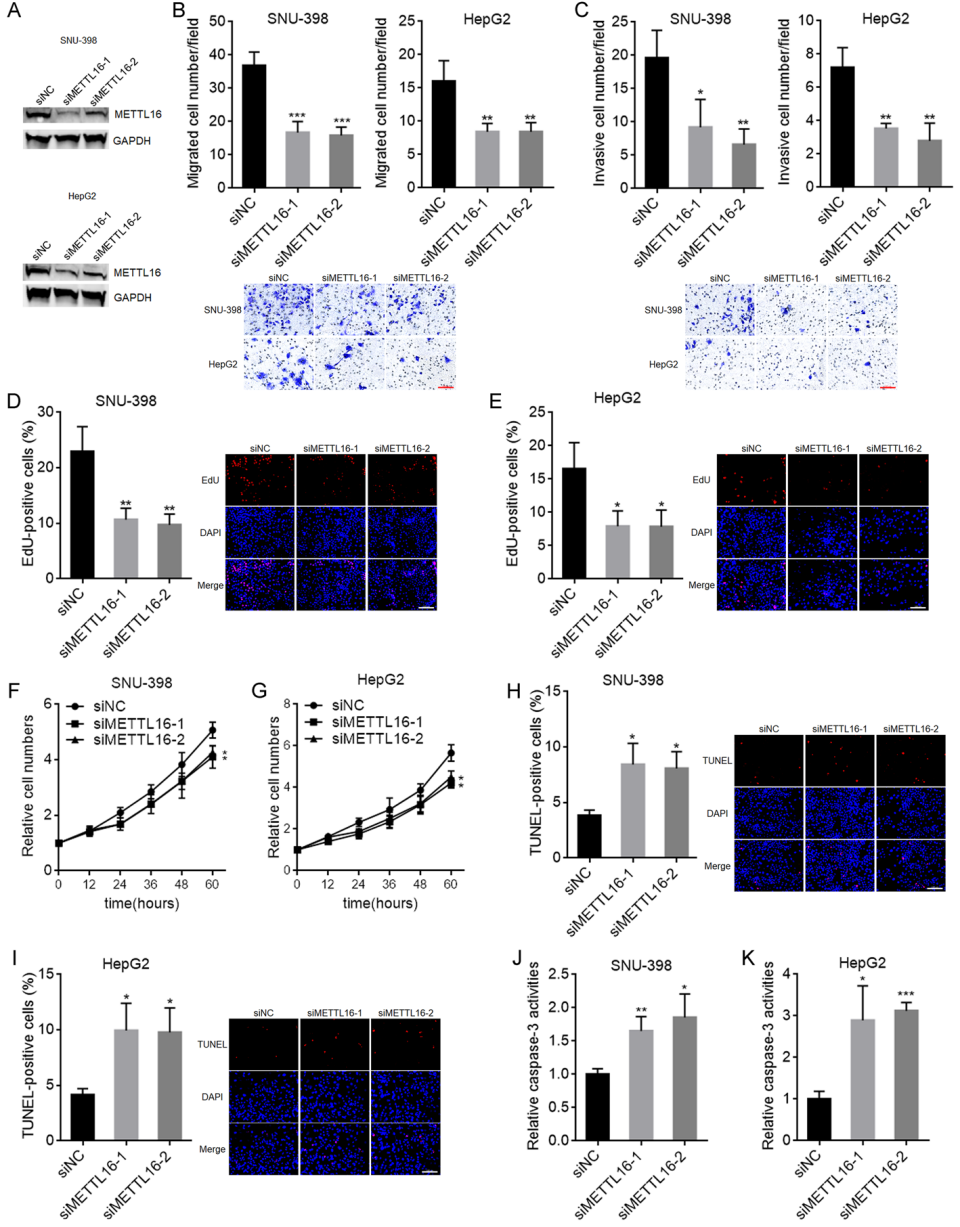
The roles of METTL16 knockdown in HCC. **A** METTL16 protein expression in SNU-398 and HepG2 cells with METTL16 knockdown or control was detected by western blot. **B** Migration ability of SNU-398 and HepG2 cells with METTL16 knockdown or control was detected by transwell migration assay. Scale bars, 100 µm. **C** Invasion ability of SNU-398 and HepG2 cells with METTL16 knockdown or control was detected by transwell invasion assay. Scale bars, 100 µm. **D** Cellular proliferation of SNU-398 cells with METTL16 knockdown or control was detected by EdU assay. Scale bars, 100 µm. **E** Cellular proliferation of HepG2 cells with METTL16 knockdown or control was detected by EdU assay. Scale bars, 100 µm. **F** Cellular proliferation of SNU-398 cells with METTL16 knockdown or control was detected by CCK-8 assay. **G** Cellular proliferation of HepG2 cells with METTL16 knockdown or control was detected by CCK-8 assay. **H** Cellular apoptosis of SNU-398 cells with METTL16 knockdown or control was detected by TUNEL assay. Scale bars, 100 µm. **I** Cellular apoptosis of HepG2 cells with METTL16 knockdown or control was detected by TUNEL assay. Scale bars, 100 µm. **J** Cellular apoptosis of SNU-398 cells with METTL16 knockdown or control was detected by caspase-3 activity assay. **K** Cellular apoptosis of HepG2 cells with METTL16 knockdown or control was detected by caspase-3 activity assay. Results are shown as mean ± s.d. of *n* = 3 independent experiments. *P < 0.05, **P < 0.01, ***P < 0.001 by one-way ANOVA followed by Dunnett’s multiple comparisons test

We next evaluated the potential biological roles of METTL16 downregulation in HCC. SNU-398 and HepG2 cells with METTL16 knockdown were constructed using two independent siRNAs against METTL16 (Fig. 3A). Transwell migration and invasion assays showed that METTL16 knockdown decreased HepG2 and SNU-398 cellular migration and invasion abilities (Fig. 3B, C). EdU assays showed that SNU-398 and HepG2 cells with METTL16 knockdown had decreased percentage of EdU-positive cells (Fig. 3D,E), indicating that METTL16 knockdown inhibited SNU-398 and HepG2 cellular proliferation. CCK-8 assays also showed that METTL16 knockdown inhibited SNU-398 and HepG2 cellular proliferation (Fig. 3F, G). TUNEL assays showed that SNU-398 and HepG2 cells with METTL16 knockdown had increased percentage of apoptosis-positive cells (Fig. 3H, I). Caspase-3 activity assays showed that METTL16 knockdown significantly increased caspase-3 activity in SNU-398 and HepG2 cells (Fig. 3J, K). Thus, both TUNEL and caspase-3 activity assays indicated that METTL16 knockdown promoted SNU-398 and HepG2 cellular apoptosis. Taken together, these results demonstrate that METTL16 knockdown inhibited HCC cellular migration, invasion, and proliferation, and promoted HCC cellular apoptosis, indicating that METTL16 silencing has tumor-suppressive roles in HCC.

### METTL16 down-regulated RAB11B-AS1 through inducing m^6^A modification

**Fig. 4:**
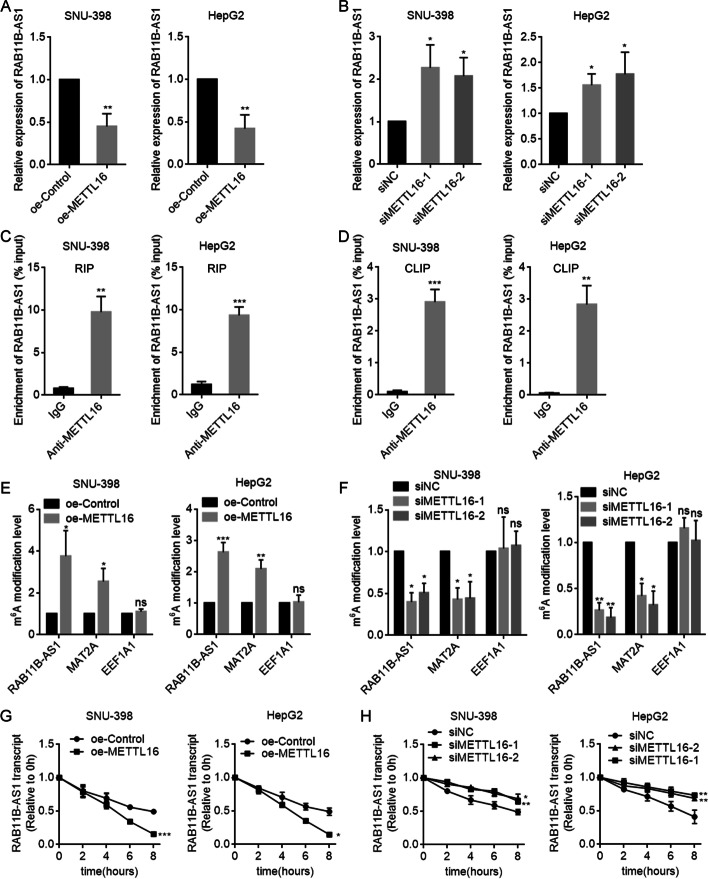
METTL16 downregulates RAB11B-AS1 through inducing m^6^A modification of RAB11B-AS1. **A** RAB11B-AS1 expression in SNU-398 and HepG2 cells with METTL16 overexpression or control was detected by RT-qPCR. **B** RAB11B-AS1 expression in SNU-398 and HepG2 cells with METTL16 knockdown or control was detected by RT-qPCR. **C** RIP assays were performed in SNU-398 and HepG2 cells to enrich the RNA bound by METTL16, followed by RT-qPCR to detect the enrichment of RAB11B-AS1. **D** CLIP assays were performed in SNU-398 and HepG2 cells to enrich the RNA directly bound by METTL16, followed by RT-qPCR to detect the enrichment of RAB11B-AS1. **E** MeRIP assays were performed in SNU-398 and HepG2 cells with METTL16 overexpression or control to enrich m^6^A modified RNA, followed by RT-qPCR to assess m^6^A modification level of RAB11B-AS1, MAT2A, and EEF1A1. **F** MeRIP assays were performed in SNU-398 and HepG2 cells with METTL16 knockdown or control to enrich m^6^A modified RNA, followed by RT-qPCR to assess m^6^A modification level of RAB11B-AS1, MAT2A, and EEF1A1. **G** The stability of RAB11B-AS1 over time was measured after blocking new RNA synthesis with α-amanitin (50 µM) in SNU-398 and HepG2 cells with METTL16 overexpression or control. **H** The stability of RAB11B-AS1 over time was measured after blocking new RNA synthesis with α-amanitin (50 µM) in SNU-398 and HepG2 cells with METTL16 knockdown or control. Results are shown as mean ± SD of *n* = 3 independent experiments. *P < 0.05, **P < 0.01, ***P < 0.001 by Student’s *t*-test (**A**, **C**, **D**, **E**, **G**) or one-way ANOVA followed by Dunnett’s multiple comparisons test (**B**, **F**, **H**)

To elucidate the underlying mechanism by which METTL16 exerted oncogenic roles in HCC, we searched the transcripts whose m^6^A modification was regulated by METTL16 through analyzing public available datasets (GSE182607 and GSE156795). We also searched the transcripts that were bound by METTL16 through analyzing public available datasets (GSE103948 and GSE156797). A total of 29 candidate transcripts bound by METTL16 were uncovered whose m^6^A modification was also regulated by METTL16. Then, we conducted RT-qPCR to evaluate the regulation of METTL16 on candidate transcripts. The results showed that RAB11B-AS1 was downregulated following METTL16 overexpression, and upregulated following METTL16 knockdown in SNU-398 and HepG2 cells (Fig. 4A, B). Next, RIP-qPCR assay suggested that RAB11B-AS1 transcript was robustly enriched by METTL16-specific antibody (Fig. 4C). CLIP assay also showed that METTL16 directly bound to RAB11B-AS1 (Fig. 4D). Furthermore, the MeRIP-qPCR assay was conducted to determine the m^6^A modification level of RAB11B-AS1. The results showed a significant increase in RAB11B-AS1 m^6^A modification level following METTL16 overexpression in SNU-398 and HepG2 cells (Fig. 4E). MAT2A, a reported target of METTL16, also had increased m^6^A modification level in this condition and was used as positive control. m^6^A modification level of EEF1A1, which was a target of METTL3, was not changed in this condition and was used as negative control. Conversely, the m^6^A modification level of RAB11B-AS1 was significantly decreased following METTL16 knockdown in SNU-398 and HepG2 cells (Fig. 4F). m^6^A modification has been frequently reported to regulate transcript stability [14]. Therefore, we further evaluated RAB11B-AS1 stability using α-amanitin to block new RNA synthesis and then measured the loss of RAB11B-AS1. The results showed that METTL16 overexpression shortened the half-life of RAB11B-AS1 (Fig. 4G). In contrast, METTL16 knockdown elongated the half-life of RAB11B-AS1 (Fig. 4H). Collectively, these data suggest that METTL16 bound RAB11B-AS1 and induced m^6^A modification of RAB11B-AS1, which reduced RAB11B-AS1 stability.

### The expression of RAB11B-AS1 was downregulated and inversely correlated with METTL16 in HCC

**Fig. 5:**
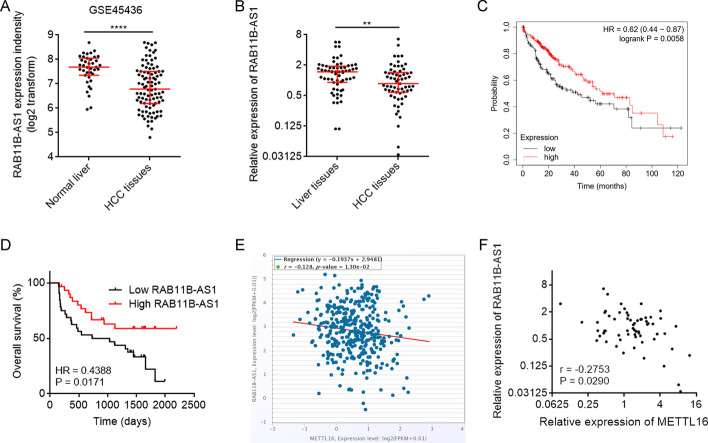
The expression pattern of RAB11B-AS1 in HCC and its association with METTL16. **A** RAB11B-AS1 expression in human HCC tissues (*n* = 95) and normal liver tissues (*n* = 39) from GSE45436 dataset. ****P < 0.0001 by Mann–Whitney test. **B** RAB11B-AS1 expression level in 63 pairs of HCC tissues and matched adjacent noncancerous liver tissues was measured by RT-qPCR. **P < 0.01 by Wilcoxon signed-rank test. **C** Kaplan–Meier survival analysis of the correlation between RAB11B-AS1 expression and overall survival based on TCGA liver cancer data, analyzed by the online in silico tool Kaplan–Meier Plotter (https://kmplot.com/analysis/). **D** Kaplan–Meier survival analysis of the correlation between RAB11B-AS1 expression and overall survival in our HCC cohort. *n* = 63 patients with HCC. *P* = 0.0171 by log-rank test. Median RAB11B-AS1 level was used as cutoff. **E** The correlation between RAB11B-AS1 and METTL16 expression in 374 HCC tissues according to TCGA dataset, analyzed by the online in silico tool ENCORI. **F** The correlation between RAB11B-AS1 and METTL16 expression in 63 HCC tissues detected by qRT-PCR. *r* = −0.2753, *P* = 0.029 by Spearman correlation analysis

The expression of RAB11B-AS1 in HCC was analyzed using GSE45436 dataset, which showed that RAB11B-AS1 was downregulated in HCC tissues compared with in normal liver tissues (Fig. 5A). In our cohort, the expression RAB11B-AS1 in HCC tissues was also lower than that in matched adjacent noncancerous liver tissues (Fig. 5B). Analysis of the TCGA dataset using the online in silico tool Kaplan–Meier Plotter showed that low RAB11B-AS1 expression level was significantly correlated with poor overall survival in HCC (Fig. 5C; Additional file 1: Fig. S2). In our cohort, Kaplan–Meier survival analysis also showed that patients with lower RAB11B-AS1 level had worse overall survival (Fig. 5D). Moreover, analysis of the TCGA dataset using the online in silico tool ENCORI showed that the expression of RAB11B-AS1 was negatively correlated with METTL16 in HCC tissues (Fig. 5E). The negative correlation between RAB11B-AS1 and METTL16 expression in HCC tissues was also confirmed in our cohort (Fig. 5F).

### Overexpression of RAB11B-AS1 exerted tumor-suppressive roles in HCC

**Fig. 6:**
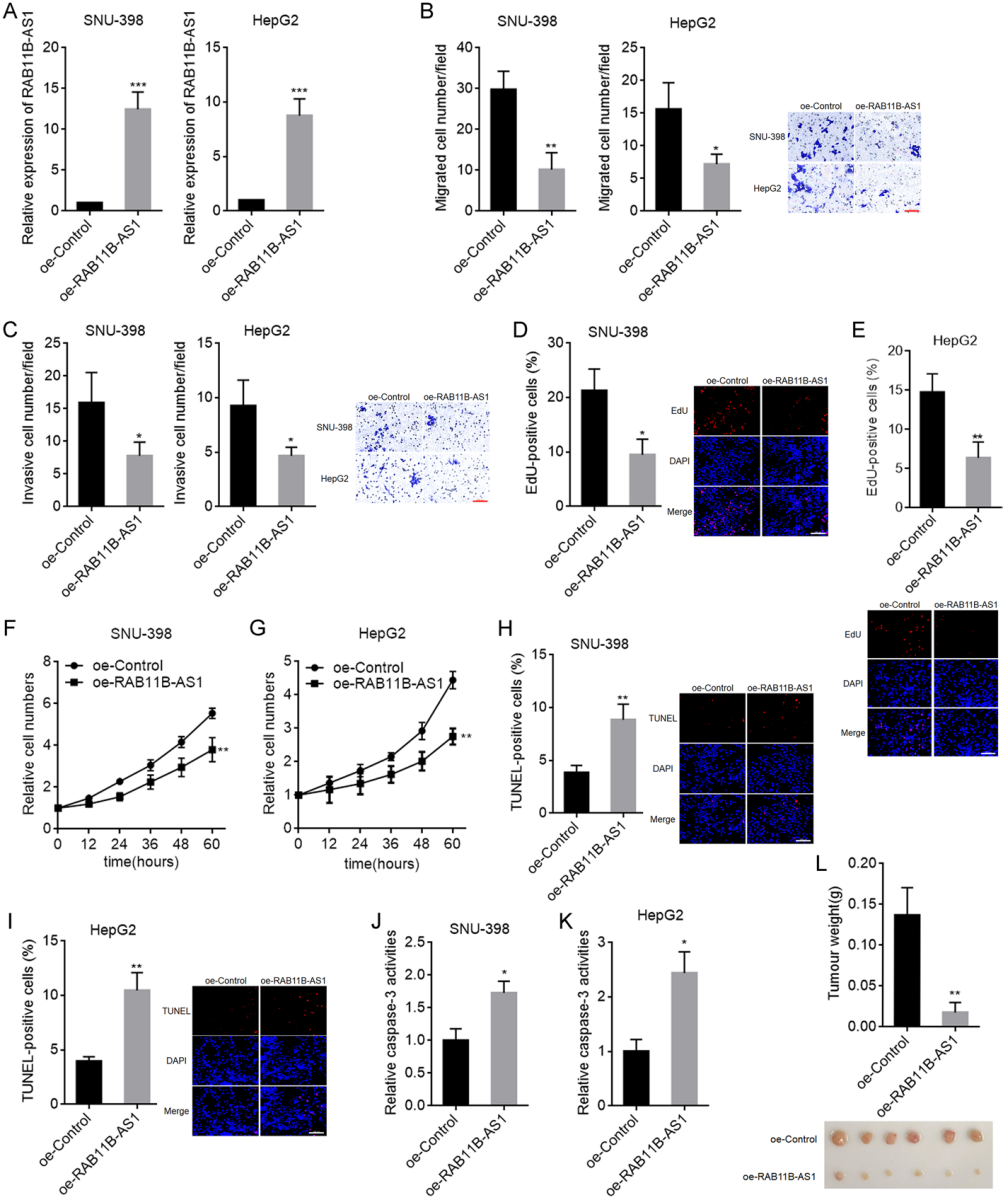
The roles of RAB11B-AS1 overexpression in HCC. **A** RAB11B-AS1 expression in SNU-398 and HepG2 cells with RAB11B-AS1 overexpression or control was detected by RT-qPCR. **B** Migration ability of SNU-398 and HepG2 cells with RAB11B-AS1 overexpression or control was detected by transwell migration assay. Scale bars, 100 µm. **C** Invasion ability of SNU-398 and HepG2 cells with RAB11B-AS1 overexpression or control was detected by transwell invasion assay. Scale bars, 100 µm. **D** Cellular proliferation of SNU-398 cells with RAB11B-AS1 overexpression or control was detected by EdU assay. Scale bars, 100 µm. **E** Cellular proliferation of HepG2 cells with RAB11B-AS1 overexpression or control was detected by EdU assay. Scale bars, 100 µm. **F** Cellular proliferation of SNU-398 cells with RAB11B-AS1 overexpression or control was detected by CCK-8 assay. **G** Cellular proliferation of HepG2 cells with RAB11B-AS1 overexpression or control was detected by CCK-8 assay. **H** Cellular apoptosis of SNU-398 cells with RAB11B-AS1 overexpression or control was detected by TUNEL assay. Scale bars, 100 µm. **I** Cellular apoptosis of HepG2 cells with RAB11B-AS1 overexpression or control was detected by TUNEL assay. Scale bars, 100 µm. **J** Cellular apoptosis of SNU-398 cells with RAB11B-AS1 overexpression or control was detected by caspase-3 activity assay. **K** Cellular apoptosis of HepG2 cells with RAB11B-AS1 overexpression or control was detected by caspase-3 activity assay. **L** Weight and photograph of subcutaneous tumors formed by SNU-398 cell with RAB11B-AS1 overexpression or control. Results are shown as mean ± SD of *n* = 3 independent experiments (**B**–**K**) or *n* = 6 mice in each group (**L**). *P < 0.05, **P < 0.01, ***P < 0.001 by Student’s *t*-test (**A**–**K**) or Mann–Whitney test (**L**)

To explore the biological roles of RAB11B-AS1 in HCC, SNU-398 and HepG2 cells with RAB11B-AS1 stable overexpression were constructed using RAB11B-AS1 overexpression plasmid (Fig. 6A). Transwell migration and invasion assays showed that RAB11B-AS1 overexpression decreased SNU-398 and HepG2 cellular migration and invasion abilities (Fig. 6B, C). EdU assays showed that SNU-398 and HepG2 cells with RAB11B-AS1 overexpression had decreased percentage of EdU-positive cells (Fig. 6D, E), indicating reduced cell proliferation. CCK-8 assays also showed that RAB11B-AS1 overexpression remarkably inhibited SNU-398 and HepG2 cellular proliferation (Fig. 6F, G). TUNEL assays showed that SNU-398 and HepG2 cells with RAB11B-AS1 overexpression had increased percentage of TUNEL-positive cells (Fig. 6H, I), indicating increased cell apoptosis. Caspase-3 activity assays showed that RAB11B-AS1 overexpression significantly increased caspase-3 activity in SNU-398 and HepG2 cells (Fig. 6J, K), also indicating increased cell apoptosis. SNU-398 cells with RAB11B-AS1 stable overexpression or control were subcutaneously injected into BALB/c nude mice. The results showed that SNU-398 cells with RAB11B-AS1 overexpression formed much smaller tumors than control SNU-398 cells (Fig. 6L). Collectively, these data demonstrate that overexpression of RAB11B-AS1 inhibited HCC cellular migration, invasion, and proliferation, promoted HCC cellular apoptosis, and inhibited HCC tumoral growth in vivo, indicating that overexpression of RAB11B-AS1 has tumor-suppressive roles in HCC.

### RAB11B-AS1 silencing exerted oncogenic roles in HCC

**Fig. 7:**
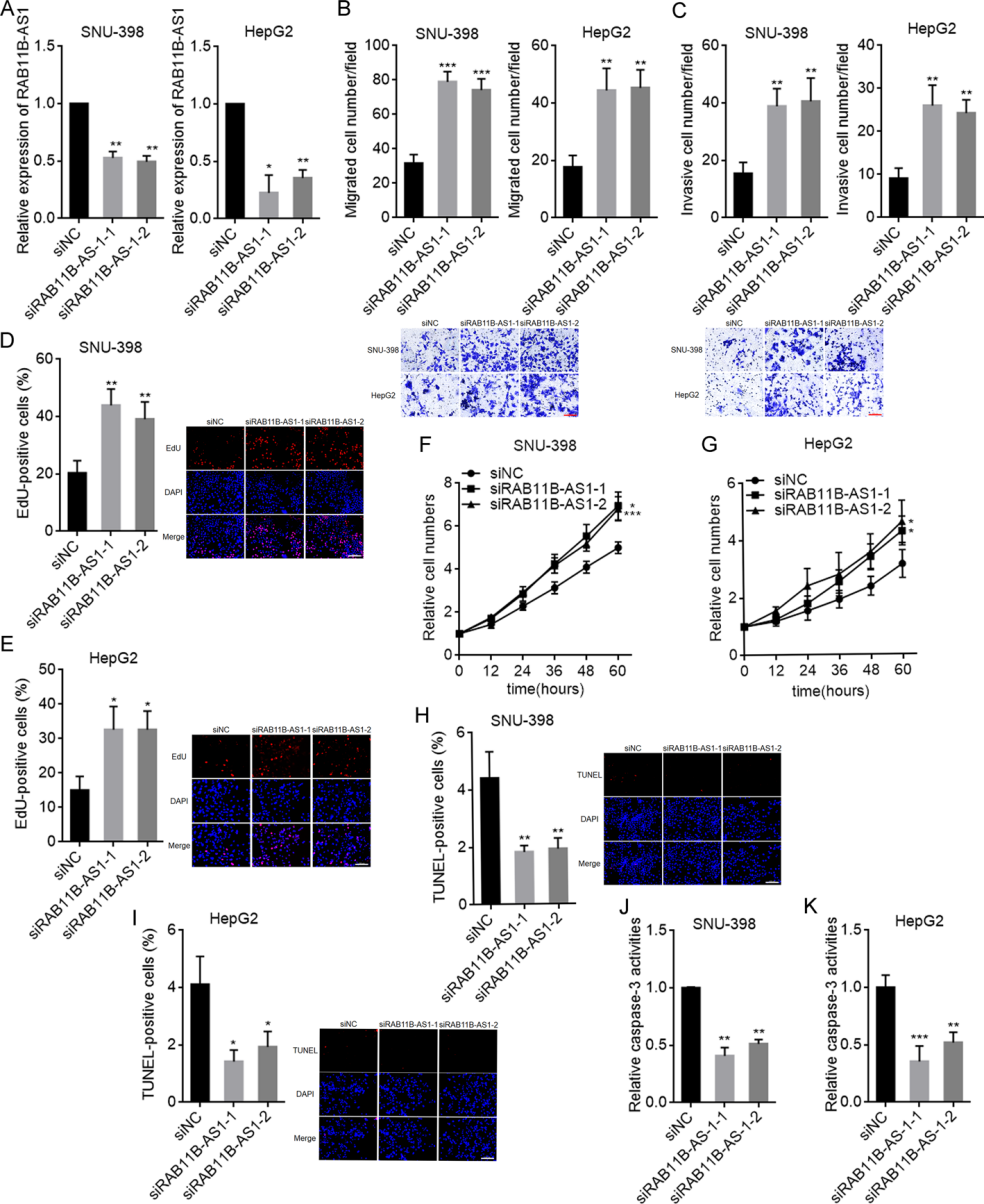
The roles of RAB11B-AS1 knockdown in HCC. **A** RAB11B-AS1 expression in SNU-398 and HepG2 cells with RAB11B-AS1 knockdown or control was detected by RT-qPCR. **B** Migration ability of SNU-398 and HepG2 cells with RAB11B-AS1 knockdown or control was detected by transwell migration assay. Scale bars, 100 µm. **C** Invasion ability of SNU-398 and HepG2 cells with RAB11B-AS1 knockdown or control was detected by transwell invasion assay. Scale bars, 100 µm. **D** Cellular proliferation of SNU-398 cells with RAB11B-AS1 knockdown or control was detected by EdU assay. Scale bars, 100 µm. **E** Cellular proliferation of HepG2 cells with RAB11B-AS1 knockdown or control was detected by EdU assay. Scale bars, 100 µm. **F** Cellular proliferation of SNU-398 cells with RAB11B-AS1 knockdown or control was detected by CCK-8 assay. **G** Cellular proliferation of HepG2 cells with RAB11B-AS1 knockdown or control was detected by CCK-8 assay. **H** Cellular apoptosis of SNU-398 cells with RAB11B-AS1 knockdown or control was detected by TUNEL assay. Scale bars, 100 µm. **I** Cellular apoptosis of HepG2 cells with RAB11B-AS1 knockdown or control was detected by TUNEL assay. Scale bars, 100 µm. **J** Cellular apoptosis of SNU-398 cells with RAB11B-AS1 knockdown or control was detected by caspase-3 activity assay. **K** Cellular apoptosis of HepG2 cells with RAB11B-AS1 knockdown or control was detected by caspase-3 activity assay. Results are shown as mean ± SD of *n* = 3 independent experiments. *P < 0.05, **P < 0.01, ***P < 0.001 by one-way ANOVA followed by Dunnett’s multiple comparisons test

Next, we investigated the potential biological roles of RAB11B-AS1 knockdown in HCC. SNU-398 and HepG2 cells with RAB11B-AS1 knockdown were constructed using two independent siRNAs against RAB11B-AS1 (Fig. 7A). Transwell migration and invasion assays showed that RAB11B-AS1 knockdown increased SNU-398 and HepG2 cellular migration and invasion abilities (Fig. 7B, C). Both EdU and CCK-8 assays showed that RAB11B-AS1 knockdown remarkably promoted SNU-398 and HepG2 cellular proliferation (Fig. 7D–G). TUNEL and caspase-3 activity assays showed that RAB11B-AS1 knockdown significantly decreased SNU-398 and HepG2 cellular apoptosis (Fig. 7H–K). Collectively, these results demonstrate that RAB11B-AS1 knockdown promoted HCC cellular migration, invasion, and proliferation, and inhibited HCC cellular apoptosis, indicating that RAB11B-AS1 silencing has oncogenic roles in HCC.

### RAB11B-AS1 reversed the oncogenic roles of METTL16 in HCC

**Fig. 8:**
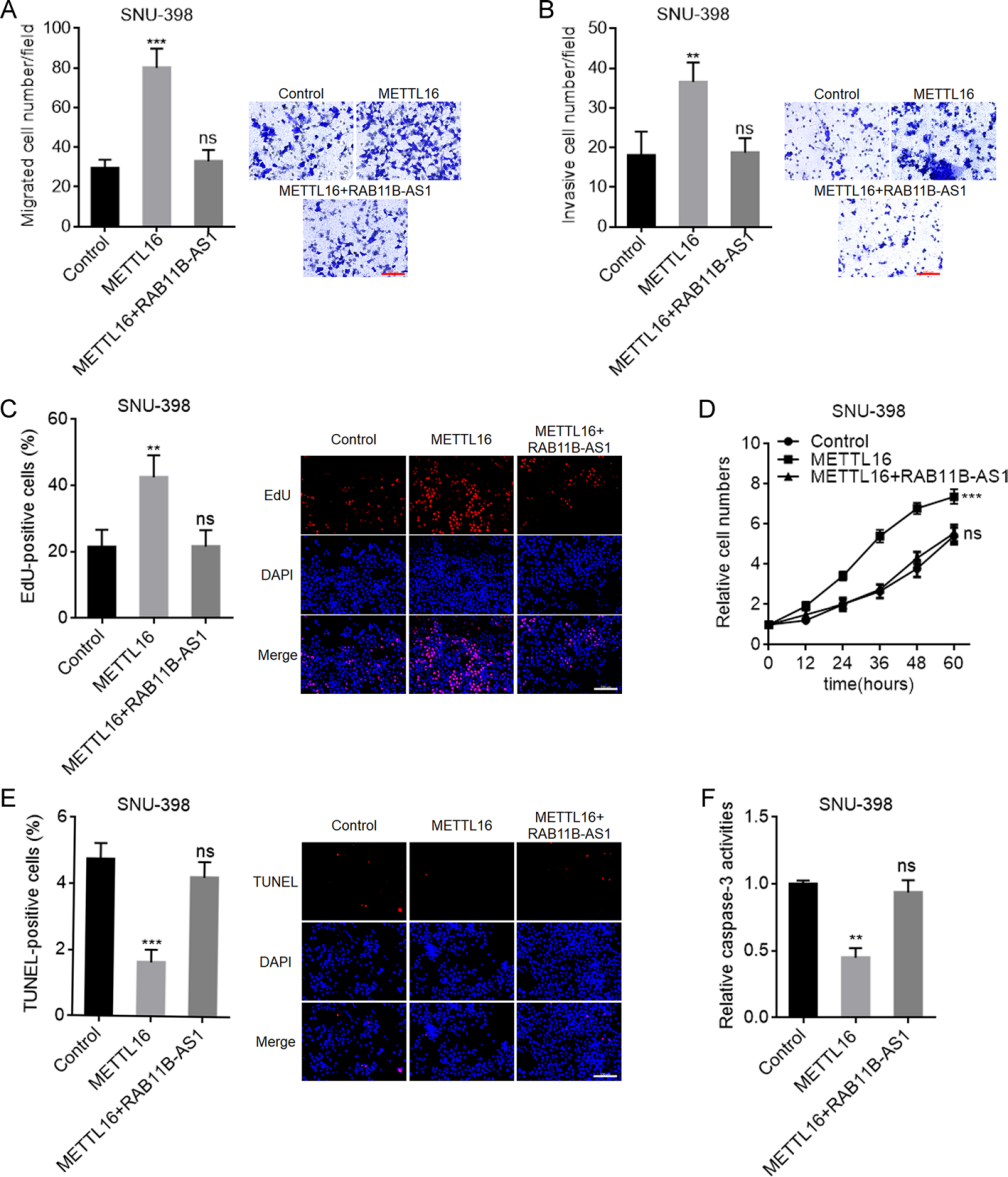
RAB11B-AS1 reverses the oncogenic roles of METTL16 in HCC. **A** Migration ability of SNU-398 cells with METTL16 and RAB11B-AS1 concurrent overexpression or control was detected by transwell migration assay. Scale bars, 100 µm. **B** Invasion ability of SNU-398 cells with METTL16 and RAB11B-AS1 concurrent overexpression or control was detected by transwell invasion assay. Scale bars, 100 µm. **C** Cellular proliferation of SNU-398 cells with METTL16 and RAB11B-AS1 concurrent overexpression or control was detected by EdU assay. Scale bars, 100 µm. **D** Cellular proliferation of SNU-398 cells with METTL16 and RAB11B-AS1 concurrent overexpression or control was detected by CCK-8 assay. **E** Cellular apoptosis of SNU-398 cells with METTL16 and RAB11B-AS1 concurrent overexpression or control was detected by TUNEL assay. Scale bars, 100 µm. **F** Cellular apoptosis of SNU-398 cells with METTL16 and RAB11B-AS1 concurrent overexpression or control was detected by caspase-3 activity assay. Results are shown as mean ± SD of *n* = 3 independent experiments. **P < 0.01, ***P < 0.001; ns, not significant, by one-way ANOVA followed by Dunnett’s multiple comparisons test

To explore whether RAB11B-AS1 was the downstream mediator of the roles of METTL16 in HCC, we stably overexpressed RAB11B-AS1 in SNU-398 and HepG2 cells with METTL16 stable overexpression. Transwell migration and invasion assays showed that RAB11B-AS1 overexpression reversed the roles of METTL16 overexpression in promoting SNU-398 and HepG2 cellular migration and invasion (Fig. 8A, B; Additional file 1: Fig. S3A, B). EdU and CCK-8 assays showed that RAB11B-AS1 overexpression reversed the roles of METTL16 overexpression in promoting SNU-398 and HepG2 cellular proliferation (Fig. 8C, D; Additional file 1: Fig. S3C, D). TUNEL and caspase-3 activity assays showed that RAB11B-AS1 overexpression reversed the roles of METTL16 overexpression in inhibiting SNU-398 and HepG2 cellular apoptosis (Fig. 8E, F; Additional file 1: Fig. S3E, F). Therefore, these data show that the oncogenic roles of METTL16 in HCC were reversed by RAB11B-AS1, and suggest that the downregulation of RAB11B-AS1 at least partially mediated the oncogenic roles of METTL16 in HCC.

## Discussion

Increasing evidence has revealed the critical roles of m^6^A modification in a variety of pathophysiological processes. Aberrant m^6^A modifications have been frequently found in various diseases and contribute to their initiation and progression [7]. Most m^6^A methyltransferases and demethylases have been intensively investigated in cancers, such as METTL3, METTL14, FTO, and ALKBH5. As a methyltransferase, METTL16 was reported to install only MAT2A and U6 snRNA with m^6^A, although METTL16 was revealed to bind to various targets [26, 51]. The involvement of METTL16 in cancers is largely unknown. Cong et al. reported that METTL16 was upregulated in glioma [52]. Liu et al. reported that METTL16 was associated with poor prognosis of patients with melanoma [53]. Wang et al. found that METTL16 promoted cell proliferation in gastric cancer [22].

In this study, we identified METTL16 as an HCC-related m^6^A methyltransferase. Public datasets, including GEO and TCGA, and our own HCC cohort both revealed that METTL16 was upregulated in HCC and its upregulation was correlated with poor prognosis of patients with HCC. Gain- and loss-of-function assays revealed that METTL16 promoted HCC cellular proliferation, migration, and invasion, inhibited HCC cellular apoptosis, and promoted HCC tumoral growth in vivo. Thus, METTL16 had oncogenic roles in HCC. Our findings suggest METTL16 as a potential prognostic and therapeutic target for HCC.

m^6^A modification exerts various effects on target RNAs, including RNA maturation, stability, and translation [7, 11, 54]. In this study, through analyzing the METTL16-bound transcripts and METTL16-regulated m^6^A differentially modified transcripts, we focused on the lncRNA RAB11B-AS1. In HCC cells, we further verified that RAB11B-AS1 was a critical downstream target of METTL16. METLL16 directly bound to RAB11B-AS1, induced m^6^A modification of RAB11B-AS1, decreased RAB11B-AS1 transcript stability, and therefore downregulated RAB11B-AS1 transcript level. Conversely to METTL16, RAB11B-AS1 was downregulated in HCC, and its low expression was correlated with poor prognosis of patients with HCC. Moreover, the expression of RAB11B-AS1 was negatively correlated with that of METTL16 in HCC tissues, supporting the negative regulation of RAB11B-AS1 by METTL16. The mechanism underlying the regulation of RAB11B-AS1 transcript stability by RAB11B-AS1 m^6^A modification needs further investigation. Nonetheless, the regulation of RAB11B-AS1 transcript stability by m^6^A modification provides novel evidence for the effects of m^6^A modification on RNA stability.

RAB11B-AS1 is a recently reported cancer-related lncRNA [47]. However, the expression and roles of RAB11B-AS1 in different tumors are not consistent [46, 47, 55, 56]. RAB11B-AS1 was reported to be induced by hypoxia and promote angiogenesis and metastasis in breast cancer via recruiting RNA polymerase II to enhance expression of angiogenic factors [46]. Li et al. reported that RAB11B-AS1 was upregulated in lung cancer, associated with poor prognosis, and promoted lung cancer metastasis via upregulating RAB11B [56]. However, RAB11B-AS1 was also found to be downregulated in osteosarcoma and suppress osteosarcoma progression via downregulating RAB11B [47]. Jiang et al. reported that RAB11B-AS1 was correlated with good prognosis in endometrial cancer [57]. In head and neck cancer, RAB11B-AS1 was also reported to be correlated with good prognosis [55]. In this study, we found that RAB11B-AS1 was downregulated in HCC and its expression was correlated with good prognosis of patients with HCC, which was confirmed by both public datasets and our own cohort. In HCC, we further found that RAB11B-AS1 exerted tumor-suppressive roles in HCC. The different roles of RAB11B-AS1 in different cancers imply that different mechanisms mediate the specific roles of RAB11B-AS1 in specific cancers. The specific mechanism mediating the tumor-suppressive roles of RAB11B-AS1 in HCC needs further exploration. Functional rescue assays revealed that the downregulation of RAB11B-AS1 mediated the oncogenic roles of METTL16 in HCC.

## Conclusions

In summary, our findings reveal that the RNA methyltransferase METTL16 was upregulated in HCC, associated with poor prognosis of patients with HCC, and promoted HCC progression via downregulating RAB11B-AS1. METTL16 directly bound to RAB11B-AS1 and installed RAB11-AS1 with m^6^A, leading to decreased stability of RAB11B-AS1 transcript and downregulation of RAB11B-AS1 transcript level. These data suggest that the METTL16–RAB11B-AS1 regulation axis represents a potential prognostic biomarker and therapeutic target for HCC.

## Supplementary Information

**Additional file 1: Fig. S1.** Output of the correlation between METTL16 expression and overall survival based on TCGA LIHC dataset analyzed by the online in silico tool Kaplan–Meier Plotter (https://kmplot.com/analysis/index.php?p=service&cancer=pancancer_rnaseq). **Fig. S2. **Output of the correlation between RAB11B-AS1 expression and overall survival based on TCGA LIHC dataset analyzed by the online in silico tool Kaplan–Meier Plotter (https://kmplot.com/analysis/index.php?p=service&cancer=pancancer_rnaseq). **Fig. S3. **RAB11B-AS1 reverses the oncogenic roles of METTL16 in HepG2 cells. (A) Migration ability of HepG2 cells with METTL16 and RAB11B-AS1 concurrent overexpression or control was detected by transwell migration assay. Scale bars, 100 µm. (B) Invasion ability of HepG2 cells with METTL16 and RAB11B-AS1 concurrent overexpression or control was detected by transwell invasion assay. Scale bars, 100 µm. (C) Cellular proliferation of HepG2 cells with METTL16 and RAB11B-AS1 concurrent overexpression or control was detected by EdU assay. Scale bars, 100 µm. (D) Cellular proliferation of HepG2 cells with METTL16 and RAB11B-AS1 concurrent overexpression or control was detected by CCK-8 assay. (E) Cellular apoptosis of HepG2 cells with METTL16 and RAB11B-AS1 concurrent overexpression or control was detected by TUNEL assay. Scale bars, 100 µm. (F) Cellular apoptosis of HepG2 cells with METTL16 and RAB11B-AS1 concurrent overexpression or control was detected by caspase-3 activity assay. Results are shown as mean ± SD of *n* = 3 independent experiments. *P < 0.05, **P < 0.01; ns, not significant, by one-way ANOVA followed by Dunnett’s multiple comparisons test.

## Data Availability

The data presented in this study are available on reasonable request from the corresponding author.

## Abbreviations

HCC: Hepatocellular carcinoma
m^6^A: *N*^6^-methyladenosine
METTL16: Methyltransferase-like protein 16
lncRNA: Long noncoding RNA
snRNA: Small nuclear RNA
FBS: Fetal bovine serum
qPCR: Quantitative polymerase chain reaction
RT: Reverse transcription
siRNA: Small interfering RNA
NC: Negative control
CCK-8: Cell Counting Kit-8
EdU: 5-Ethynyl-2′-deoxyuridine
TUNEL: Terminal deoxynucleotidyl transferase (TdT)-mediated dUTP nick end labeling
RIP: RNA immunoprecipitation
GEO: Gene expression omnibus
TCGA: The Cancer Genome Atlas
MeRIP: Methylated RNA immunoprecipitation
