# Supplementary material for: METTL16 promotes hepatocellular carcinoma progression through downregulating RAB11B-AS1 in an m6A-dependent manner
Source: Cell Mol Biol Lett. 2022 May 20;27:41. doi: 10.1186/s11658-022-00342-8 (PMC9123709; doi:10.1186/s11658-022-00342-8)

**Additional Information**

**Additional file 1: Fig. S1** Output of the correlation between METTL16 expression and overall survival based on TCGA LIHC dataset analyzed by the online in silico tool Kaplan-Meier Plotter (https://kmplot.com/analysis/index.php?p=service&cancer=pancancer_rnaseq). **
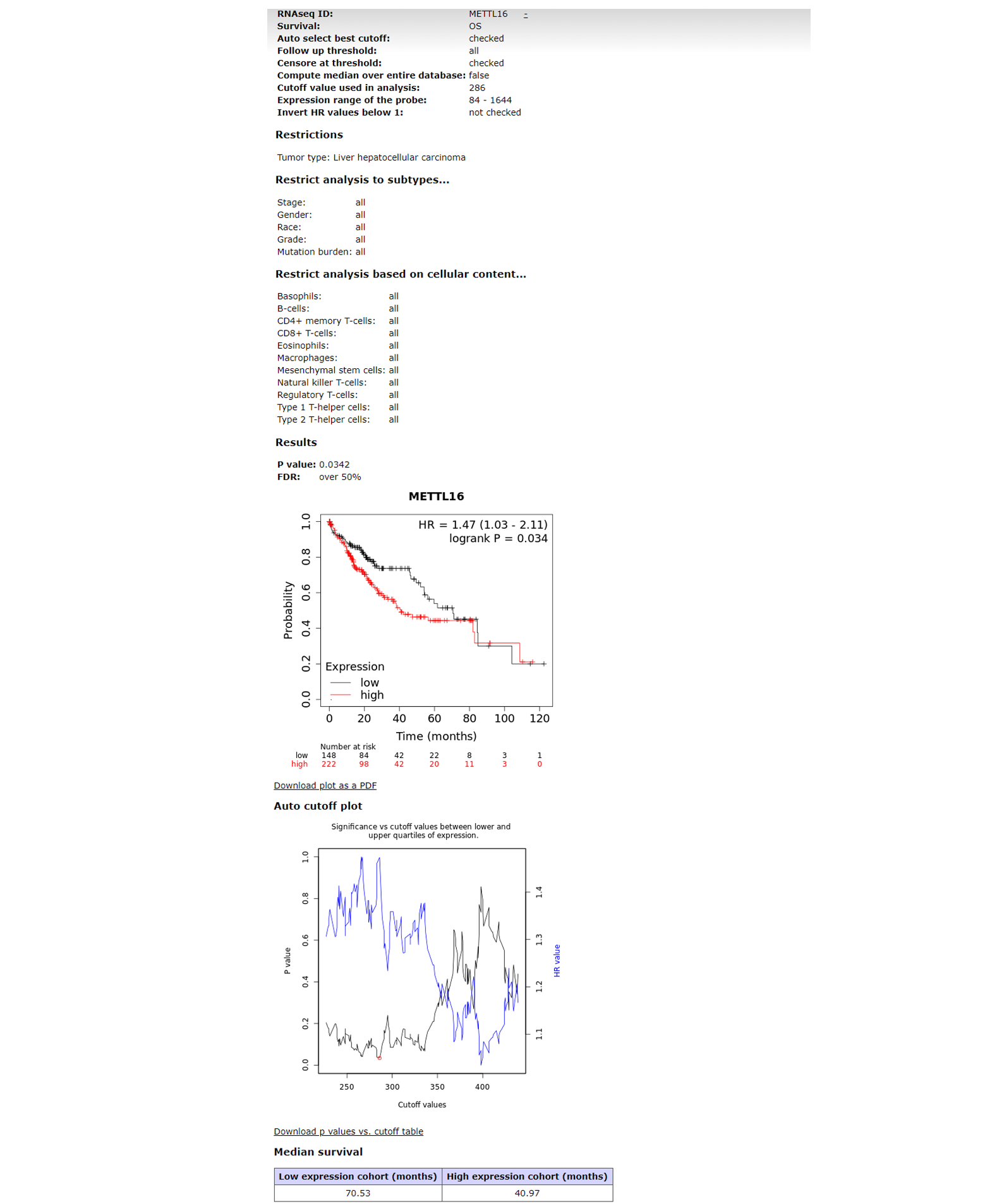
**

**Fig. S2** Output of the correlation between RAB11B-AS1 expression and overall survival based on TCGA LIHC dataset analyzed by the online in silico tool Kaplan-Meier Plotter (<https://kmplot.com/analysis/index.php?p=service&cancer=pancancer_rnaseq>).


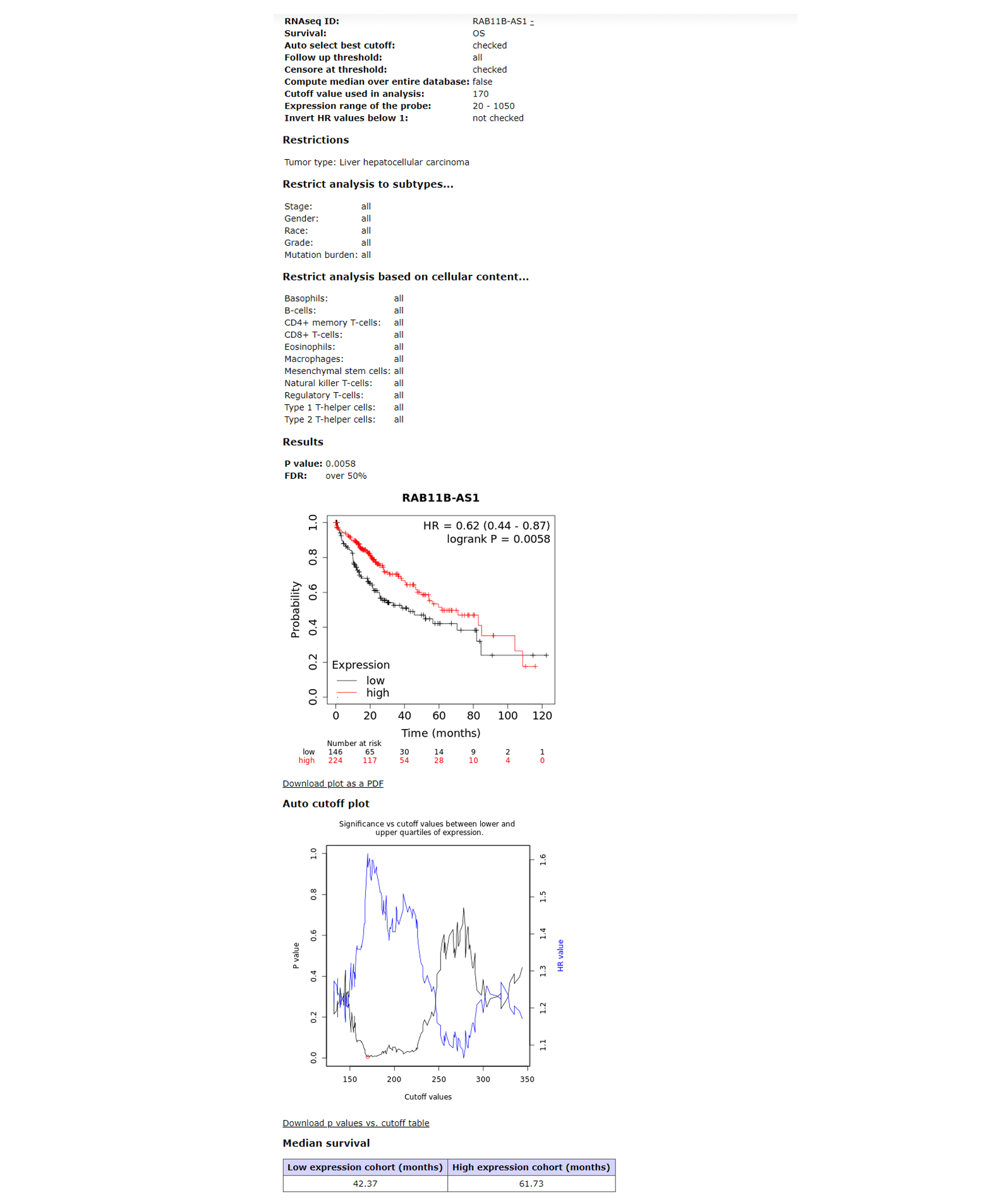


**Fig. S3** RAB11B-AS1 reverses the oncogenic roles of METTL16 in HepG2 cells. **A** Migration ability of HepG2 cells with METTL16 and RAB11B-AS1 concurrent overexpression or control was detected by transwell migration assay. Scale bars = 100 µm. **B** Invasion ability of HepG2 cells with METTL16 and RAB11B-AS1 concurrent overexpression or control was detected by transwell invasion assay. Scale bars = 100 µm. **C** Cellular proliferation of HepG2 cells with METTL16 and RAB11B-AS1 concurrent overexpression or control was detected by EdU assay. Scale bars = 100 µm. **D** Cellular proliferation of HepG2 cells with METTL16 and RAB11B-AS1 concurrent overexpression or control was detected by CCK-8 assay. **E** Cellular apoptosis of HepG2 cells with METTL16 and RAB11B-AS1 concurrent overexpression or control was detected by TUNEL assay. Scale bars = 100 µm. **F** Cellular apoptosis of HepG2 cells with METTL16 and RAB11B-AS1 concurrent overexpression or control was detected by caspase-3 activity assay. Results are shown as mean ± s.d. of n = 3 independent experiments. *P < 0.05, **P < 0.01, ns, not significant, by one-way ANOVA followed by Dunnett's multiple comparisons test.


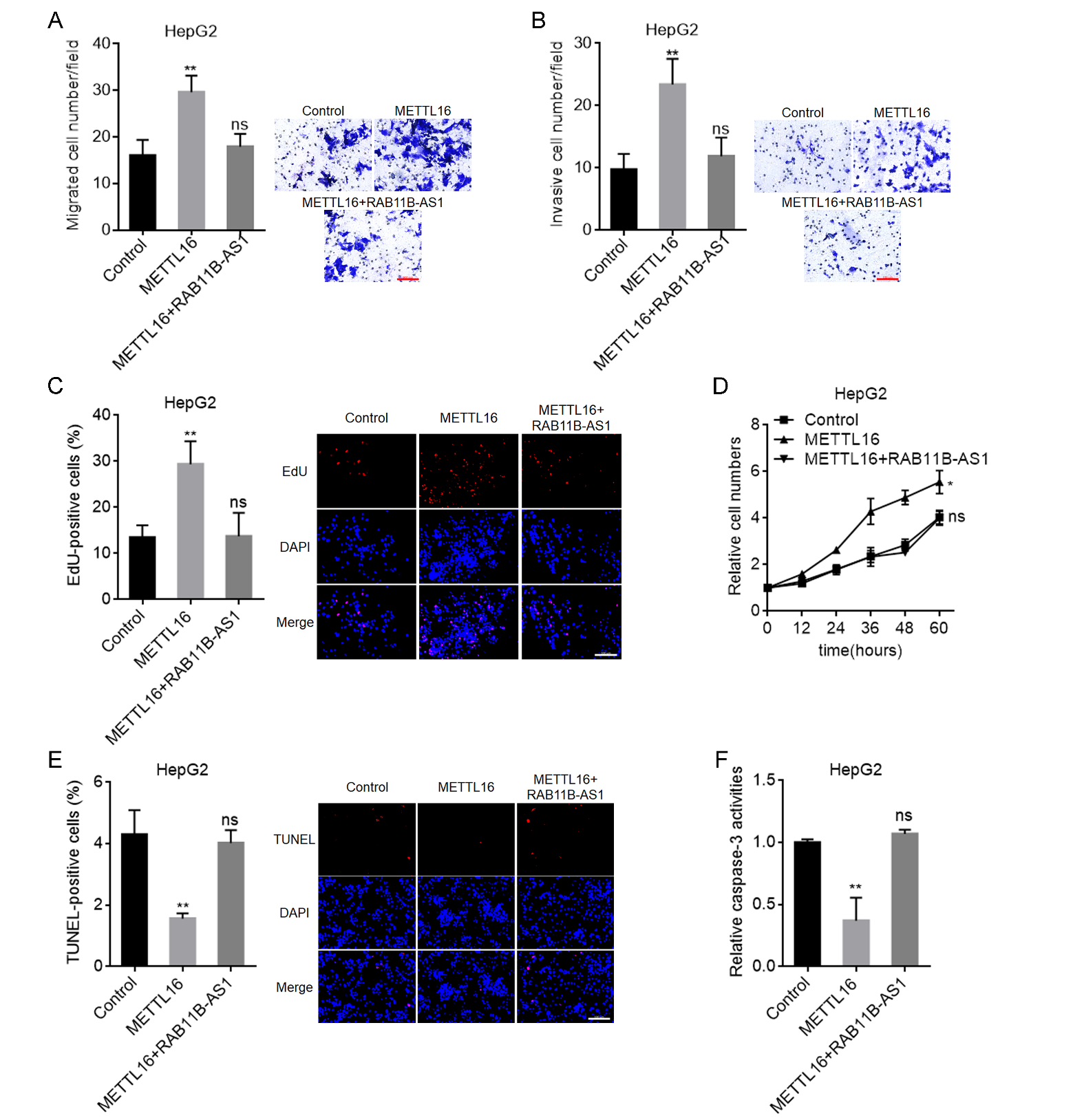

Supplement: Supplementary file 1 — Additional file 1: Fig. S1. Output of the correlation between METTL16 expression and overall survival based on TCGA LIHC dataset analyzed by the online in silico tool Kaplan–Meier Plotter (https://kmplot.com/analysis/index.php?p=service&cancer=pancancer_rnaseq). Fig. S2. Output of the correlation between RAB11B-AS1 expression and overall survival based on TCGA LIHC dataset analyzed by the online in silico tool Kaplan–Meier Plotter (https://kmplot.com/analysis/index.php?p=service&cancer=pancancer_rnaseq). Fig. S3. RAB11B-AS1 reverses the oncogenic roles of METTL16 in HepG2 cells. (A) Migration ability of HepG2 cells with METTL16 and RAB11B-AS1 concurrent overexpression or control was detected by transwell migration assay. Scale bars, 100 µm. (B) Invasion ability of HepG2 cells with METTL16 and RAB11B-AS1 concurrent overexpression or control was detected by transwell invasion assay. Scale bars, 100 µm. (C) Cellular proliferation of HepG2 cells with METTL16 and RAB11B-AS1 concurrent overexpression or control was detected by EdU assay. Scale bars, 100 µm. (D) Cellular proliferation of HepG2 cells with METTL16 and RAB11B-AS1 concurrent overexpression or control was detected by CCK-8 assay. (E) Cellular apoptosis of HepG2 cells with METTL16 and RAB11B-AS1 concurrent overexpression or control was detected by TUNEL assay. Scale bars, 100 µm. (F) Cellular apoptosis of HepG2 cells with METTL16 and RAB11B-AS1 concurrent overexpression or control was detected by caspase-3 activity assay. Results are shown as mean ± SD of n = 3 independent experiments. *P < 0.05, **P < 0.01; ns, not significant, by one-way ANOVA followed by Dunnett’s multiple comparisons test. [file 11658_2022_342_MOESM1_ESM.docx]
